# Supplementary figures and images for: Ecological Packaging: Reuse and Recycling of Rosehip Waste to Obtain Biobased Multilayer Starch-Based Material and PLA for Food Trays
Source: Foods. 2025 May 22;14(11):1843. doi: 10.3390/foods14111843 (PMC12154451; doi:10.3390/foods14111843)

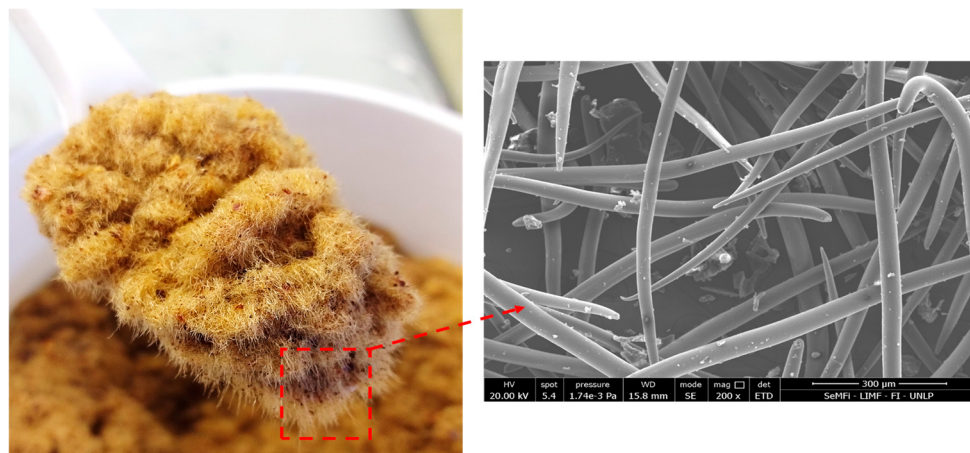

**Figure S1.** Photographs of rosehip oil extraction residue and SEM micrographs.

Supplement: Supplementary file 1 [file foods-14-01843-s001.zip › foods-3616247-supplementary.pdf]
